# Hyperglycemia alters enzyme activity and cell number in spinal sensory ganglia

**DOI:** 10.1186/1749-7221-2-11

**Published:** 2007-04-25

**Authors:** Richard A Zaruba, Paul N Epstein, Patrick A Carr

**Affiliations:** 1 Department of Anatomy and Cell Biology, School of Medicine and Health Sciences, University of North Dakota, Grand Forks, ND 58202, USA; 2 Department of Pediatrics, University of Louisville, Louisville, KY 40202, USA

## Abstract

Peripheral sensory diabetic neuropathy is characterized by morphological, electrophysiological and neurochemical changes to a subpopulation of primary afferent neurons. Here, we utilized a transgenic mouse model of diabetes (OVE26) and age-matched controls to histologically examine the effect of chronic hyperglycemia on the activity or abundance of the enzymes acid phosphatase, cytochrome oxidase and NADPH-diaphorase in primary sensory neuron perikarya and the dorsal horn of the spinal cord. Quantitative densitometric characterization of enzyme reaction product revealed significant differences between diabetic, compared to control, animals for all three enzymes. Levels of acid phosphatase reaction product were found to be significantly reduced in both small diameter primary sensory somata and the dorsal horn. Cytochrome oxidase activity was found to be significantly lower in small primary sensory somata while NADPH-diaphorase labeling was found to be significantly higher in small primary sensory somata and significantly lower in the dorsal horn. In addition to these observed biochemical changes, ratiometric analysis of the number of small versus large diameter primary sensory perikarya in diabetic and control animals demonstrated a quantifiable decrease in the number of small diameter cells in the spinal ganglia of diabetic mice. These results suggest that the OVE26 model of diabetes mellitus produces an identifiable disturbance in specific metabolic pathways of select cells in the sensory nervous system and that this dysfunction may reflect the progression of a demonstrated cell loss.

## Background

Diabetic sensory neuropathies are a common, clinically observed sequelae of hyperglycemia and are characterized by a progressive degradation of primary afferent function [1,2]. Functional and structural evidence suggest an early and frequent involvement of small diameter primary sensory neurons leading to nociceptive abnormalities [2-4]. In order to examine basic mechanisms underlying this disorder, we utilized the OVE26 transgenic mouse model of diabetes mellitus [5,6] to examine the effects of long-standing hyperglycemia on enzyme histochemical indicators of sensory neuron metabolism and evaluate the potential utility of this model for future studies of diabetic neuropathy. The OVE26 mouse line uses cell-specific overexpression of calmodulin to destroy pancreatic β-cells and the result is a viable diabetic mouse (>1 year survival) that displays both early-onset (<1 week after birth) and chronic elevated blood glucose (>500 mg/dl) and decreased serum and pancreatic insulin (<50% of normal) [5,6]. Enzyme histochemical techniques demonstrated to be sensitive to neuronal perturbation [7] were used to examine the impact of long-standing hyperglycemia and hypoinsulinemia on the distribution and activity of lysosomal acid β-glycerophosphatase (AP), cytochrome oxidase (CO), and NADPH-diaphorase (NADPH-d; a correlate of nitric oxide synthase in aldehyde fixed tissue [8]) in both sensory ganglia and the spinal cord. It has been previously demonstrated [7,11] that the metabolic status of sensory neurons, as reflected by the endogenous activity of specific homeostatic enzymes, is sensitive to injury and perturbation. Therefore, these enzymes were selected as putatively reflective of mitochondrial function (CO), lysosomal or degradative activity (AP) and primary sensory neuron injury or repair (NADPH-diaphorase).

The OVE26 transgenic mouse line (characterized by the insulin promoter-linked overexpression of calmodulin in pancreatic β-cells) used in this study displays a well-characterized chronic hyperglycemia and hypoinsulinemia within days after birth. [5,6]. Ten (five OVE26 transgenic and five age-matched, control FVB animals) aged (>365 days old) mice were anesthetized with pentobarbital, perfused with 4% paraformaldehyde and the lumbar spinal cord and sensory ganglia removed, sectioned and processed for AP, CO or NADPH-d enzyme histochemistry as previously described [7,9-12]. Counts of primary sensory somata were conducted on toluidine blue counterstained sections of L5 spinal ganglia and quantified using previously published methodologies [7,12].

Quantitative analysis of CO, AP and NADPH-d staining was undertaken on both the dorsal horn of the L5 segment of the spinal cord and the large and small cells of L5 sensory ganglion using previously described densitometric analysis [7,12]. The entire mediolateral extent of lamina I to III was selected for staining intensity measurement. Statistical analyses (*t*-test, Mann-Whitney Rank Sum test, one way analysis of variance, Kruskal-Wallis analysis of variance on ranks, z-test of proportions) were conducted using SigmaStat (Jandel). Controls for densitometric analysis consisted of: 1) simultaneous sectioning and mounting of diabetic and control tissue on the same slide to ensure identical histological processing; 2) statistical analysis to verify consistency of staining between animals within control and experimental groups; 3) correction for small fluctuations in tissue opacity/thickness by subtractive illumination whereby the density value of white matter was subtracted from the immediately adjacent ventral horn; and 4) manual adjustment and calibration of the video camera parameters and microscope illumination and acquisition of all images using identical settings. All experiments were conducted in accordance with the guidelines of our institutions and the National Institutes of Health regarding the care and use of animals for experimental procedures.

**Figure 1:**
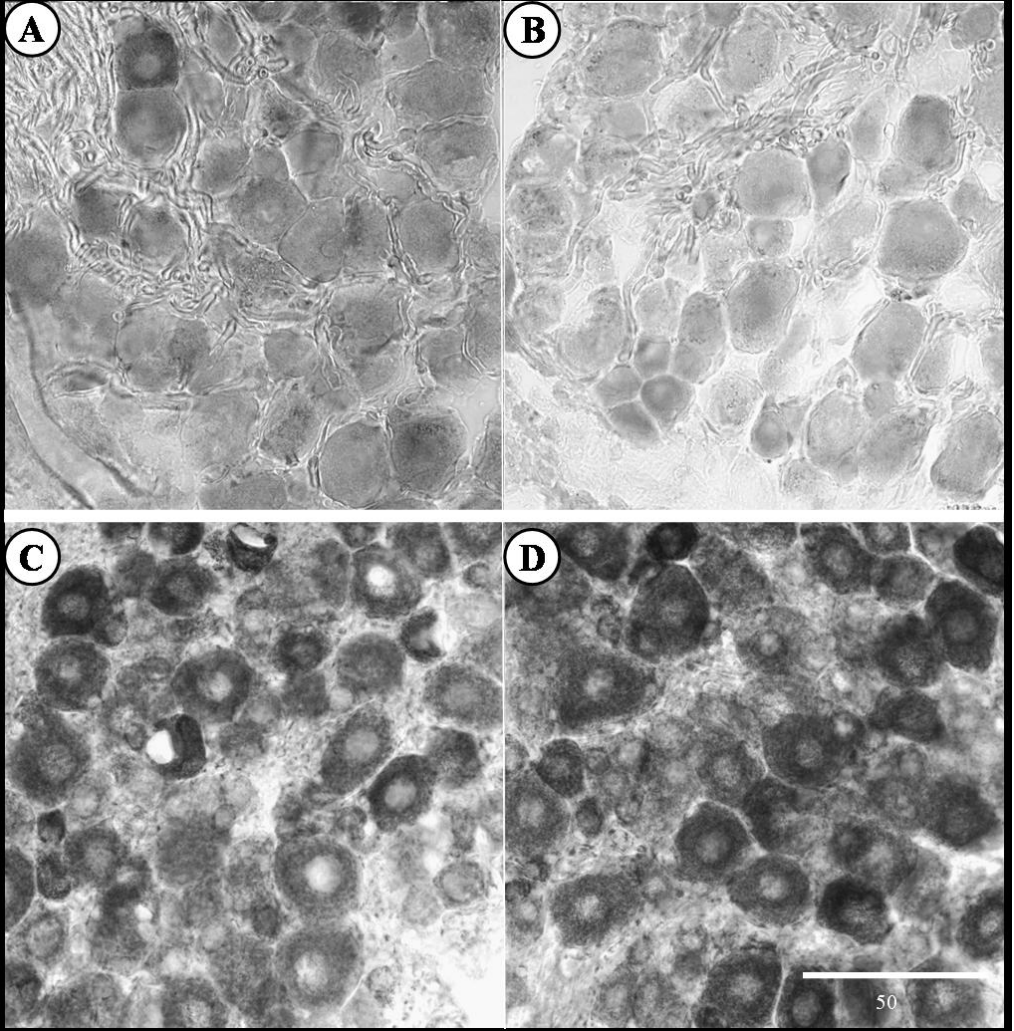
Enzyme histochemical reaction product in the fifth lumbar dorsal root ganglion from control and diabetic mice. (A,B) Cytochrome oxidase reaction product in sections from control (A) and the diabetic (B) mice. Diabetic mice display reduced levels of CO reaction product compared to control. Quantitative analysis revealed a decrease in CO reaction product density in small neuronal somata. (C,D) NADPH-diaphorase reaction product in sections from control (C) and diabetic (D) mice. Diabetic mice display an increase in NADPH-diaphorase reaction product density compared to control. Scale bar in microns.

Prior to fixative perfusion, the phenotypic status of OVE26 diabetic mice were confirmed by their characteristic small eyes caused by the GR19 gene in their transgenic construct [5]. All adult OVE26 mice maintained fed blood glucose levels of at least 400 mg/dl. At the histological level, a survey [13] of the ratio of small (50 and 500 μm^2 ^area) to large (500 and 1950 μm^2 ^area) primary sensory somata in the fifth lumbar spinal ganglia revealed a significant decrease in the proportion of small to large cells in diabetic (1.29:1 small:large perikarya) compared to control (1.94:1 small:large perikarya) mice (*P *< 0.05 by *z*-test of proportions; 417 cells measured). Quantitative densitometric analysis of the abundance and distribution of enzyme histochemical reaction product in dorsal root ganglia (DRG) revealed substantive differences between diabetic and control mice (720 cells were quantified for both densitometry and cell size; 240 cells for each enzyme). Small somata from the ganglia of diabetic mice exhibited lower levels of AP (13.4% decrease; *P *< 0.001) and CO (Fig 1A,B; 9% decrease; *P *< 0.001) reaction product and an increase in the density of the reaction product for NADPH-d (Fig. 1C,D; 13.2% increase; *P *< 0.001) in comparison to control animals. No differences were observed in large diameter neurons from diabetic as compared to control animals.

**Figure 2:**
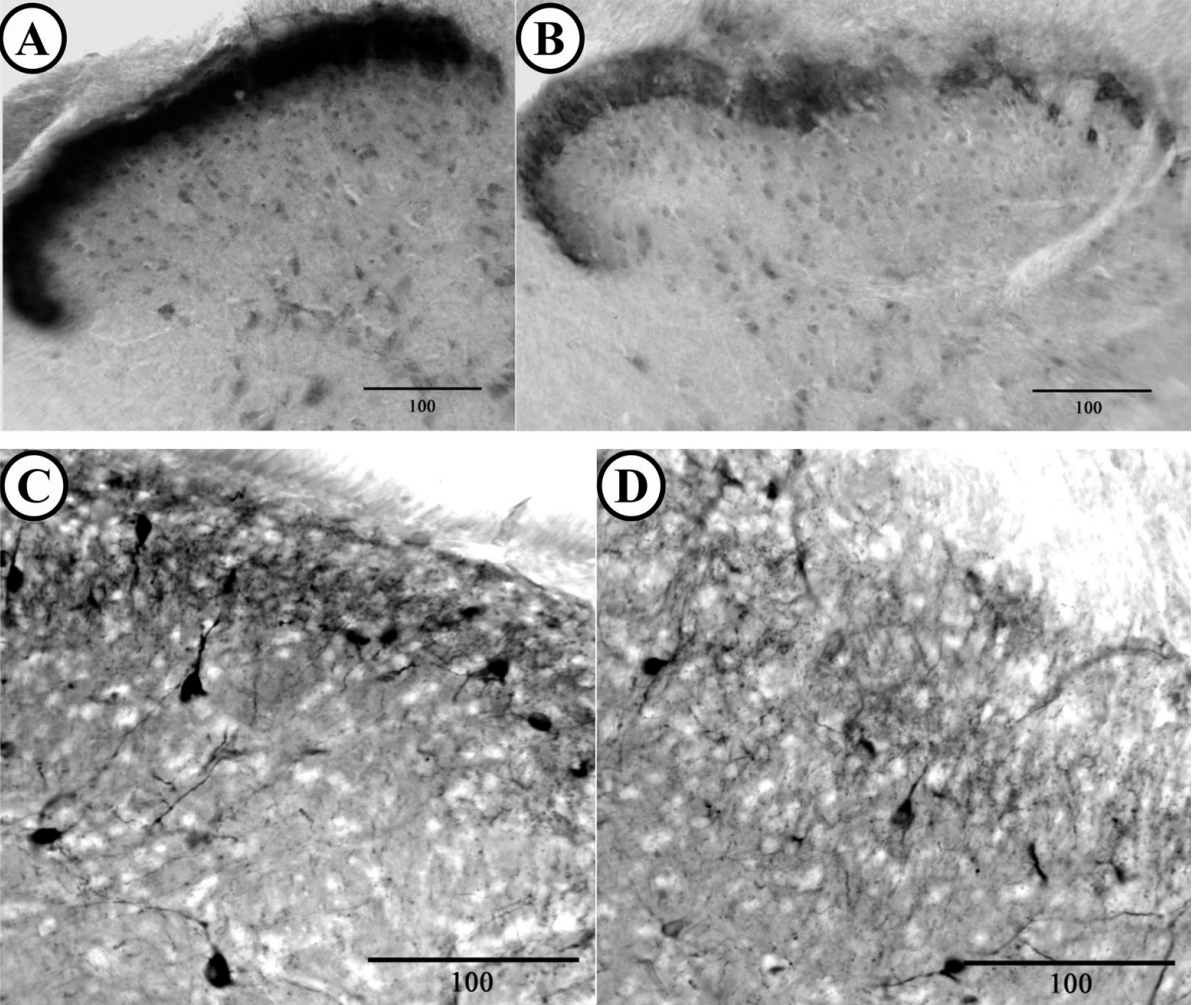
Enzyme histochemical reaction product in the dorsal horn of the fifth lumbar spinal cord from control and diabetic mice. (A,B) Acid phosphatase reaction product in sections from control (A) and the diabetic (B) mice. Diabetic mice display reduced levels of AP reaction product in medial lamina I and II of the dorsal horn. (C,D) NADPH-diaphorase reaction product in lamina I and II of control (C) and diabetic (D) mice. A reduction in the number of labeled fibers and somata can be observed in the superficial dorsal horn of diabetic animals. Scale bar in microns and dorsal horn is to top in all sections.

In the spinal cord, all observed differences were confined to lamina I to III. Motoneuron somata in the ventral horn appeared both qualitatively and quantitatively similar in diabetic and control animals. In diabetic animals, there was an observable loss of AP reaction product in lamina I and II of the dorsal horn (Fig. 2A) as compared to control mice (Fig. 2B). Similarly, these laminae appeared to have qualitatively fewer NADPH-d labeled fibers and neuronal somata in diabetic (Fig. 2C) as compared to control animals. (Fig. 2D). The decrease of both AP and NADPH-d labeling was most profound in the medial portion of lamina I and II. Quantitative densitometric analysis supported the qualitative observations and revealed significantly reduced levels of AP (*P *= 0.026; 27 sections quantified) and NADPH-d (*P *< 0.001; 27 sections quantified) reaction product in lamina I and II of the dorsal horn of control and diabetic mice (Table 1). No significant differences were observed in qualitative staining appearance or intensity of CO reaction product labeling in the dorsal horn of diabetic, compared to non-diabetic animals (31 sections quantified).

**Table 1:** Quantitative histochemical reaction product in the dorsal horn of control and diabetic mice.

Enzyme	Control Mean ± S.D.	Diabetic Mean ± S.D	*t*-test significance level
AP	6.058 ± 0.254	5.949 ± 0.210	P = 0.026*
CO	3.997 ± 0.172	3.989 ± 0.137	P = 0.800
NADPH-d	1.320 ± 0.354	0.733 ± 0.228	P < 0.001*

* indicates significant difference. Standard deviation (S.D.). Following densitometric measurements, the numbers were transformed to a 0 to 10 scale for clarity of presentation and comparison. Optical density values fall along a range of 0 representing minimal staining intensity (no staining) and 10 representing extremely dense staining (black).

Here we have demonstrated that chronic hyperglycemia has an impact on both the survival and metabolic profile of primary sensory neurons. The observed decrease in the ratio of small to large diameter primary sensory somata in diabetic animals most likely represents a loss of unmyelinated or small myelinated primary sensory neurons although a relative increase in the number of large myelinated neurons, however, unlikely, cannot be discounted. Nonetheless, the former interpretation is supported by the observed decrease in AP labeling in the dorsal horn of the spinal cord. The observed decrease in AP intensity in the surviving small neuronal somata from the DRG of the OVE26 animals, as compared to small neurons from control DRG, suggests that acid phosphatase activity in those cells is depressed. As FRAP containing sensory neurons represent a subpopulation of unmyelinated C-fibers [14], our results suggest that there is a loss, or at least a metabolic disruption of, unmyelinated neurons in the dorsal horn and in DRG. These results are consistent with the possibility of apoptosis and cell loss in the DRG of rodent models of diabetes [15,16] although differences in model, species and duration of hyperglycemia must be considered [17,18] along with the likelihood that there are a spectrum of different diabetic neuropathies including painful (small fiber involvement) and non-painful (large-fiber involvement) syndromes [2,3]. In light of this, it should not be entirely unexpected that our observations of a putative small fiber disorder complements findings of large fiber disorders in alternate animal models of diabetes [19].

Cytochrome oxidase is the terminal enzyme in the electron transport chain, and is therefore considered to be a strong indicator of somatic mitochondrial activity. The decrease in CO staining within small DRG neurons, as compared to a similar cohort of small neurons from control animal ganglia, suggests a disruption of oxidative metabolism which corresponds well with results from other animal models of diabetes that demonstrate diminished CO activity or disruptions in mitochondrial morphology or function [16,20-22]. Alternatively, our observed decrease in CO staining may reflect a simple decrease in mitochondrial number, as DRG neurons exposed to high glucose *in vitro *exposure contain fewer mitochondria [23]. The lack of an observed change in CO activity in the dorsal horn is not unexpected as both physical (axotomy) and functional (tetrodotoxin) disconnection have previously been shown to leave CO activity in the dorsal horn unaltered [7].

In DRG and the dorsal horn of the spinal cord, NADPH-d activity levels have been previously shown to be responsive to peripheral neuronal injury or attenuation of electrical activity [7]. Our results suggest that in addition to the pathological state that led to our observed loss of sensory neurons (and diminished NADPH-d labeling in the dorsal horn), there is a ongoing perturbation resulting in increased NADPH-d labeling in small DRG neurons from hyperglycemic animals as compared to the primary sensory somata from normoglycemic animals. As utilized here, NADPH-d enzyme histochemical reaction product represents nitric oxide synthase activity [8]. The elevated ganglionic NADPH-diaphorase and diminished CO labeling in the DRG of hyperglycemic mice is consistent with previously proposed inhibition of CO activity [24] by the product of nitric oxide synthase, nitric oxide. Although the reported statistically significant changes may appear to be quantitatively modest, these percent changes represent group averages. Qualitatively and quantitatively, the changes are more pronounced in some animals and tissues sections, and less obvious in others. This is not unexpected as chronic diseases processes impact individuals with profound variability in both severity and temporal progress.

Our results suggest that the OVE26 model of chronic hyperglycemia does alter the overall neurochemical profile of the sensory nervous system through cell loss and/or altered enzyme activity and that this pathology seems to specifically impact unmyelinated and/or small myelinated primary sensory neurons.

## Declaration of competing interests

The author(s) declare that they have no competing interests.

## Authors' contributions

RZ completed this work as part of his doctoral dissertation and was involved in the writing of this manuscript and contributed both intellectually and practically to the content. PE created, characterized and supplied the transgenic mice and was also involved in the writing of this manuscript and contributed both intellectually and practically to the content. PC provided the lab, supervision, and support for this work, exclusive of that associated with generation and characterization of the mouse model. PC was also involved in the design and coordination of this study and participated in the writing of this manuscript and contributed both intellectually and practically to the content. All authors read and approved the final manuscript.
